# Comparative Genomics of *Campylobacter fetus* from Reptiles and Mammals Reveals Divergent Evolution in Host-Associated Lineages

**DOI:** 10.1093/gbe/evw146

**Published:** 2016-06-22

**Authors:** Maarten J. Gilbert, William G. Miller, Emma Yee, Aldert L. Zomer, Linda van der Graaf-van Bloois, Collette Fitzgerald, Ken J. Forbes, Guillaume Méric, Samuel K. Sheppard, Jaap A. Wagenaar, Birgitta Duim

**Affiliations:** ^1^Department of Infectious Diseases and Immunology, Faculty of Veterinary Medicine, Utrecht University, the Netherlands; ^2^US Department of Agriculture, Produce Safety and Microbiology Research Unit, Agricultural Research Service, Albany, California; ^3^WHO Collaborating Center for Campylobacter/OIE Reference Laboratory for Campylobacteriosis, Utrecht, the Netherlands; ^4^Biotechnology Core Facility Branch, Division of Scientific Resources, National Center for Emerging and Zoonotic Infectious Diseases, CDC, Atlanta, Georgia; ^5^School of Medicine and Dentistry, University of Aberdeen, United Kingdom; ^6^College of Medicine, Institute of Life Science, Swansea University, United Kingdom; ^7^MRC Cloud-Based Infrastructure for Microbial Bioinformatics (CLIMB) Centre, Swansea University, United Kingdom; ^8^Department of Zoology, University of Oxford, United Kingdom; ^9^Central Veterinary Institute of Wageningen UR, Lelystad, the Netherlands

**Keywords:** *campylobacter fetus*, reptile, mammal, comparative genomics, recombination, evolution

## Abstract

*Campylobacter fetus* currently comprises three recognized subspecies, which display distinct host association. *Campylobacter fetus* subsp. *fetus* and *C*. *fetus* subsp. *venerealis* are both associated with endothermic mammals, primarily ruminants, whereas *C*. *fetus* subsp. *testudinum* is primarily associated with ectothermic reptiles. Both *C. fetus* subsp. *testudinum* and *C. fetus* subsp. *fetus* have been associated with severe infections, often with a systemic component, in immunocompromised humans. To study the genetic factors associated with the distinct host dichotomy in *C. fetus*, whole-genome sequencing and comparison of mammal- and reptile-associated *C*. *fetus* was performed. The genomes of *C*. *fetus* subsp. *testudinum* isolated from either reptiles or humans were compared with elucidate the genetic factors associated with pathogenicity in humans. Genomic comparisons showed conservation of gene content and organization among *C*. *fetus* subspecies, but a clear distinction between mammal- and reptile-associated *C*. *fetus* was observed. Several genomic regions appeared to be subspecies specific, including a putative tricarballylate catabolism pathway, exclusively present in *C*. *fetus* subsp. *testudinum* strains. Within *C*. *fetus* subsp. *testudinum*, *sapA*, *sapB*, and *sapAB* type strains were observed. The recombinant locus *iamABC* (*mlaFED*) was exclusively associated with invasive *C*. *fetus* subsp. *testudinum* strains isolated from humans. A phylogenetic reconstruction was consistent with divergent evolution in host-associated strains and the existence of a barrier to lateral gene transfer between mammal- and reptile-associated *C*. *fetus*. Overall, this study shows that reptile-associated *C*. *fetus* subsp. *testudinum* is genetically divergent from mammal-associated *C*. *fetus* subspecies.

## Introduction

*Campylobacter fetus* has been recognized as a significant veterinary pathogen. Until recently, two subspecies were described: *Campylobacter fetus* subsp. *fetus* (Cff) and *Campylobacter fetus* subsp. *venerealis* (Cfv). Both subspecies have been isolated from multiple vertebrate hosts, mainly mammals, but the primary reservoir is considered to be ruminants. These subspecies display distinct host and niche preferences: Cff is often associated with the intestinal tract and aborted fetuses of ruminants, mainly sheep and cattle, whereas Cfv is almost exclusively associated with the genital tract of cattle (van Bergen et al. 2008). Next to the aforementioned subspecies, a genetically distinct variant of *C*. *fetus* has been isolated from reptiles, with a reported prevalence of 5.5–6.7%, and humans (Harvey and Greenwood 1985; Tu et al. 2004; Dingle et al. 2010 Patrick et al. 2013; Wang et al. 2013; Gilbert, Kik, et al. 2014). This reptile-associated *C*. *fetus* has been described as *C. fetus* subsp. *testudinum* (Cft) (Fitzgerald et al. 2014).

Human infections caused by Cft have been reported and a reptilian origin in these cases is suspected (Tu et al. 2004; Patrick et al. 2013). In contrast to *C*. *jejuni*, symptoms of *C. fetus*-associated gastrointestinal illness are rarely reported (Wagenaar et al. 2014). Although cases of human Cft infection can be considered rare and opportunistic, and occur mostly in immunocompromised people, the systemic component in the majority of infections makes it difficult to treat and these infections are often life-threatening.

DNA sequence-based typing, including multilocus sequence typing (MLST) and amplified fragment length polymorphism (AFLP) analysis, have shown that reptile-associated Cft are genetically distinct from mammal-associated Cff and Cfv and that genetic diversity is higher in Cft (Dingle et al. 2010; Fitzgerald et al. 2014). This suggests host-associated evolutionary divergence between mammal- and reptile-associated *C*. *fetus* (Tu et al. 2001, 2005; Dingle et al. 2010; Kienesberger et al. 2014). Furthermore, the diversity among Cft isolates from reptiles is higher than the diversity among Cft isolates from humans (Dingle et al. 2010; Fitzgerald et al. 2014), which suggests that a subset of genotypes is able to colonize and potentially infect humans or that humans are selectively exposed to a subset of the population.

The genetic factors underlying host differentiation of mammal- and reptile-associated *C*. *fetus* are poorly understood. Comparing the whole-genome sequences of mammal- and reptile-associated *C*. *fetus* strains can provide valuable insights into this distinct host association, as well as further insights into speciation, taxonomy, and pathogenicity.

In this study, 20 genomes of reptile-associated Cft strains were compared with 39 genomes of mammal-associated Cff and Cfv. To determine features specific to *C*. *fetus*, genomes of the most closely related species *Campylobacter hyointestinalis* and *Campylobacter iguaniorum* were included in the analyses. A phylogenetic reconstruction provided insights into the distinct host association of *C*. *fetus*. Furthermore, detailed genome analyses characterized genomic regions specific to *C*. *fetus*, and to Cft in particular, and revealed multiple species- and subspecies-specific sequence variations, including a distinct putative tricarballylate catabolism locus, and a genomic region associated with human invasive strains.

## Materials and Methods

### Strains and Growth Conditions

A total of 61 strains were used for this study, including: 20 Cft strains of reptilian (*n* = 13) and human (*n* = 7) origin, 39 Cff and Cfv strains of bovine (*n* = 33), ovine (*n* = 1), human (*n* = 2), and unknown (*n* = 3) origin, one *C*. *hyointestinalis* strain of porcine origin, and one *C*. *iguaniorum* strain of reptilian origin. Characteristics of all strains used in this study are shown in table 1. Strains were grown on blood agar in a microaerobic atmosphere (83.3% N_2_, 7.1% CO_2_, 3.6% H_2_, and 6% O_2_) at 37 °C for 48 h.

**Table 1:** Features of the *Campylobacter* Strains Used in This Study

Species	Strain	Host	Source	Origin	MLST	*atpA*	*sap*	*wcbK*	Sequence Method	Level (contigs)	**References**	Accession Number
Cff	04/554	Bovine	Fetus	AR	5	3	B	+	454, Illumina, PacBio	Complete	van der Graaf-van Bloois et al. 2014	CP008808–CP008809
Cff	82-40	Human	Blood	US	6	4	A	−	Sanger	Complete	Unpublished	CP000487
Cff	98/v445	Bovine	Prepuce	UK	3	1	B	+	454	Draft (192)	van der Graaf-van Bloois et al. 2014	LMBH00000000
Cff	B0042	Bovine	Feces	UK	5	3	B	+	Illumina	Draft (25)	van der Graaf-van Bloois et al. 2016	ERR419595
Cff	B0047	Bovine	Feces	UK	5	3	B	+	Illumina	Draft (16)	van der Graaf-van Bloois et al. 2016	ERR419600
Cff	B0066	Bovine	Feces	UK	3	1	B	+	Illumina	Draft (29)	van der Graaf-van Bloois et al. 2016	ERR419610
Cff	B0097	Bovine	Feces	UK	2	2	A	−	Illumina	Draft (25)	van der Graaf-van Bloois et al. 2016	ERR419623
Cff	B0129	Bovine	Feces	UK	3	1	B	+	Illumina	Draft (32)	van der Graaf-van Bloois et al. 2016	ERR419637
Cff	B0130	Bovine	Feces	UK	3	1	B	+	Illumina	Draft (26)	van der Graaf-van Bloois et al. 2016	ERR419638
Cff	B0131	Bovine	Feces	UK	6	4	A	−	Illumina	Draft (28)	van der Graaf-van Bloois et al. 2016	ERR419639
Cff	B0151	Bovine	Feces	UK	5	3	B	+	Illumina	Draft (19)	van der Graaf-van Bloois et al. 2016	ERR419648
Cff	B0152	Bovine	Feces	UK	5	3	B	+	Illumina	Draft (18)	van der Graaf-van Bloois et al. van der Graaf-van Bloois et al. 2016	ERR419649
Cff	B0167	Bovine	Feces	UK	5	3	B	+	Illumina	Draft (19)	van der Graaf-van Bloois et al. van der Graaf-van Bloois et al. 2016	ERR460866
Cff	B0168	Bovine	Feces	UK	5	3	B	+	Illumina	Draft (27)	van der Graaf-van Bloois et al. van der Graaf-van Bloois et al. 2016	ERR460867
Cff	BT 10/98	Ovine	Unknown	UK	2	2	A	−	454	Draft (44)	van der Graaf-van Bloois et al. 2014	LRAL00000000
Cff	H1-UY	Human	Blood	UY	4	1	A^a^	−	Illumina	Draft (34)	Iraola et al. 2015	JYCP00000000
Cff	S0693A	Bovine	Feces	UK	5	3	B	+	Illumina	Draft (20)	van der Graaf-van Bloois et al. van der Graaf-van Bloois et al. 2016	ERR419284
Cff	S0478D	Bovine	Feces	UK	3	1	B	+	Illumina	Draft (24)	van der Graaf-van Bloois et al. van der Graaf-van Bloois et al. 2016	ERR419653
Cft	03-427	Human	Blood	US	15	6	A	−	454, Illumina, PacBio	Complete	Gilbert et al. 2013	CP006833
Cft	11S02557-2	Chelonian (*Mauremys annamensis*)	Feces	NL	58	5	A	−	Illumina	Draft (62)	This study	LFMJ00000000
Cft	11S05168-1	Snake (*Python reticulatus*)	Feces	NL	59	5	A	−	Illumina	Draft (109)	This study	LELD00000000
Cft	12S00416-3	Chelonian (*Geochelone elegans*)	Feces	NL	60	5	B	+	Illumina	Draft (52)	This study	LFLJ00000000
Cft	12S02225-3	Lizard (*Tiliqua rugosa*)	Feces	NL	61	5	A	−	Illumina	Draft (54)	This study	LFLK00000000
Cft	12S02263-3	Chelonian *(Pseudemys* sp.)	Feces	NL	62	5	A	−	Illumina	Draft (116)	This study	LFLL00000000
Cft	12S02842-30	Chelonian (*Aldabrachelys gigantea*)	Feces	NL	63	5	B	+	Illumina	Draft (104)	This study	LFLM00000000
Cft	12S02847-1	Snake (*Boa constrictor*)	Feces	NL	64	5	AB	+	Illumina	Draft (148)	This study	LFLN00000000
Cft	12S02855-1	Snake (*Orthriophis taeniurus*)	Feces	NL	65	5	AB	+	Illumina	Draft (118)	This study	LFLO00000000
Cft	12S04217-1	Chelonian (*Cuora mouhotii*)	Feces	NL	66	5	B	+	Illumina	Draft (147)	This study	LFLP00000000
Cft	13S00388-15	Chelonian (*Chelonoidis denticulata*)	Feces	NL	67	5	A	−	Illumina	Draft (223)	This study	LFLQ00000000
Cft	85-387	Chelonian (*Terrapene carolina*)	Feces	US	16	5	AB	+	Illumina	Draft (81)	This study	LFXC00000000
Cft	CF78-2	Lizard (*Tiliqua nigrolutea*)	Feces	UK	26	5	A	−	Illumina	Draft (59)	This study	LFXD00000000
Cft	D4335	Human	Feces	US	31	5	A	−	Illumina	Draft (56)	This study	LFXE00000000
Cft	D6659	Human	Pleural fluid	US	15	6	A	−	Illumina	Draft (54)	This study	LFXF00000000
Cft	D6683	Human	Hematoma	US	15	6	A	−	Illumina	Draft (74)	This study	LFXG00000000
Cft	D6690	Human	Blood	US	15	6	A	−	Illumina	Draft (137)	This study	LMBF00000000
Cft	D6783	Human	Feces	US	30	5	A	−	Illumina	Draft (65)	This study	LMBE00000000
Cft	D6856	Human	Bile	US	15	6	A	−	Illumina	Draft (169)	This study	LMBG00000000
Cft	SP3	Snake (*Heterodon nasicus*)	Feces	UK	27	5	A	−	454, Illumina, PacBio	Complete	This study	CP010953
Cfv	02/298	Bovine	Fetus	AR	4	1	A	−	454	Draft (233)	van der Graaf-van Bloois et al. 2014	LRVK00000000
Cfv^b^	03/293	Bovine	Unknown	AR	4	1	A	−	454, Illumina, PacBio	Complete	van der Graaf-van Bloois et al. 2014	CP0006999–CP007002
Cfv	03/596	Bovine	Fetus	AR	4	1	A	−	454	Draft (103)	van der Graaf-van Bloois et al. 2014	LRAM00000000
Cfv	642-21	Bovine	Uterus	AU	4	1	A^a^	−	Illumina	Draft (126)	Barrero et al. 2014	AJSG00000000
Cfv	84-112	Bovine	Genital secretion	US	4	1	A	−	454	Complete	Kienesberger et al. 2014	HG004426–HG004427
Cfv	92/203	Bovine	Placenta	AR	4	1	A	−	454	Draft (133)	van der Graaf-van Bloois et al. 2014	LRVL00000000
Cfv	97/532	Bovine	Vagina	AR	4	1	A	−	454	Draft (125)	van der Graaf-van Bloois et al. 2014	LRER00000000
Cfv	97/608	Bovine	Placenta	AR	4	1	A	−	454, Illumina, PacBio	Complete	van der Graaf-van Bloois et al. 2014	CP008810–CP008812
Cfv	98/25	Bovine	Fetus	AR	4	1	A	−	454	Draft (100)	van der Graaf-van Bloois et al. 2014	LRES00000000
Cfv	99/541	Bovine	Prepuce	AR	4	1	A^a^	−	Illumina	Draft (218)	Iraola et al. 2013	ASTK00000000
Cfv	ADRI 513	Unknown	Unknown	AU	4	1	A	−	454	Draft (103)	van der Graaf-van Bloois et al. 2014	LRFA00000000
Cfv^b^	ADRI 1362	Bovine	Unknown	AR	4	1	A	−	454	Draft (98)	van der Graaf-van Bloois et al. 2014	LREX00000000
Cfv	B6	Bovine	Vagina	AU	4	1	A^a^	−	Illumina	Draft (81)	Barrero et al. 2014	AJMC00000000
Cfv	B10	Bovine	Unknown	US	4	1	A	−	454	Draft (133)	van der Graaf-van Bloois et al. 2014	LRET00000000
Cfv	CCUG 33872	Unknown	Abortion	FR	4	1	A	−	454	Draft (101)	van der Graaf-van Bloois et al. 2014	LREU00000000
Cfv	CCUG 33900	Bovine	Abortion	FR	4	1	A	−	454	Draft (53)	van der Graaf-van Bloois et al. 2014	LREV00000000
Cfv	LMG 6570	Bovine	Unknown	BE	4	1	A	−	454	Draft (63)	van der Graaf-van Bloois et al. 2014	LREW00000000
Cfv	NCTC 10354	Bovine	Vagina	UK	4	1	A	−	454	Draft (45)	Stynen et al. 2011	AFGH00000000
Cfv	WBT 011/09	Unknown	Unknown	UK	4	1	A	−	454	Draft (77)	van der Graaf-van Bloois et al. 2014	LMBI00000000
Cfv	Zaf 3	Bovine	Fetus	SA	4	1	A	−	454	Draft (59)	van der Graaf-van Bloois et al. 2014	LREZ00000000
Cfv^b^	Zaf 65	Bovine	Unknown	SA	4	1	A	−	454	Draft (90)	van der Graaf-van Bloois et al. 2014	LREY00000000
Chy	DSM 19053	Porcine	Intestine	US	n/a	n/a	n/a	−	IonTorrent	Draft (26)	Unpublished	JHQP00000000
Cig	1485E	Lizard (*Pogona vitticeps*)	Feces	NL	n/a	n/a	n/a	+	454, Illumina, PacBio	Complete	Gilbert, Miller, et al. 2014	CP009043–CP009044

Note.—Cig, *C*. *iguaniorum*; Chy, *C*. *hyointestinalis*. AR, Argentina; AU, Australia; BE, Belgium; CZ, Czech Republic; FR, France; NL, Netherlands; SA, South Africa; UK, United Kingdom; US, United States; UY, Uruguay. MLST, multilocus sequence typing (sequence type); *atpA*, ATP synthase alpha subunit (MLST allele number); *sap*, *sap* type (A, B, or AB); *wcbK*, GDP-mannose 4,6-dehydratase (+, present; -, absent); n/a, not applicable.

^a^Predicted *sap* type based on sequence type and the absence of *wcbK*.

^b^Subspecies designation inconclusive; subspecies *fetus* or *venerealis* based on phenotype, subspecies *venerealis* based on genotype (van der Graaf-van Bloois et al. 2014).

### Genome Sequencing

In total, 61 genomes were included in this study (table 1). The whole-genome sequence data of 39 Cff and Cfv strains were from published studies (Stynen et al. 2011; Iraola et al. 2013; Barrero et al. 2014; Kienesberger et al. 2014; van der Graaf-van Bloois et al. 2014, 2016; Iraola et al. 2015). For 18 Cft strains, Illumina MiSeq reads were generated in this study; an average of 2.18 million Illumina MiSeq reads per strain were assembled using Newbler (v2.6), resulting in draft genomes containing, on average, 31 large contigs (>5,000 bp; average contig size = 61 kb) and 338× coverage.

Whole-genome sequence data of Cft strains 03-427 and SP3 were obtained using a Roche 454 genome analyzer and was assembled into contigs using the Newbler assembler (v2.6). In this study, the genome of Cft strain SP3 was completed using methodology described previously for Cft strain 03-427 (Gilbert et al. 2013). In short, 221,254 Roche 454 reads were assembled into a single scaffold of 24 contigs using the Newbler assembler (v2.6). All Roche 454 base calls were validated using 1,852,600 Illumina MiSeq reads, providing a total coverage of 156×. Sequences across the contig junctions and the S-layer (*sap*) locus were confirmed with Sanger sequencing. Assembly was confirmed using Pacific Biosystems long reads. PacBio RS reads were assembled into contigs using Quiver (Pacific Bioscience, Menlo Park, CA).

*Campylobacter iguaniorum* strain 1485E was sequenced as described previously (Gilbert, Miller, et al. 2014). The whole-genome sequence of *C*. *hyointestinalis* strain DSM 19053 was obtained from GenBank.

The completed genomes of Cft strains 03-427 and SP3, Cff strain 82-40, and Cfv strain 97/608 were used as reference genomes.

### Genome Analysis

The genomes of Cft strains 03-427 and SP3 were annotated as described previously (Gilbert et al. 2013). Homopolymeric GC tracts were characterized using the high-depth MiSeq reads. CRISPR regions were identified using CRISPRFinder (Grissa et al. 2007). Genes were assigned a functional category using the RAST subsystem annotation approach as described (Overbeek et al. 2014). In the completed genomes, by comparison with a close relative, genes which were truncated due to a premature stop codon, frame-shifted, fragmented or had a missing start codon were defined as pseudogenes.

A local BLASTP, including all strains listed in table 1, was performed, based on the predicted proteomes of all genomes, and the results were screened for *C*. *fetus* species- and subspecies-specific features. To visualize genomic regions specific for *C*. *fetus* and *C*. *fetus* subsp. *testudinum* in particular, the BLAST ring image generator (BRIG) (Alikhan et al. 2011) was used at default settings, based on BLASTN v2.2.26. For this, the contigs of all Cft strains, Cff strain 82-40, and Cfv strain 97/608 were selected and the complete genome of Cft strain 03-427 was used as a reference.

The *sap* locus contains a high number of repeated sequences and its assembly can prove difficult. The *sap* type of the draft Cft genomes was identified by mapping the sequence reads to the primer sequences of SAF01, SAR01, SBF01, and SBR01 (Tu et al. 2001) using PASS (Campagna et al. 2009). Only exact matches to the primer sequences were considered.

### Phylogeny and Recombination Analysis

Recombination analysis of *C. fetus* was performed using Gubbins (Croucher et al. 2014). Briefly, open reading frames were predicted and annotated using Prokka (Seemann 2014) and all versus all BLAST was performed for all predicted proteins of the genomes (table 1) at an E-value cutoff of 1E-6. To determine the orthologous relationships of all proteins, the BLAST output was parsed by Orthagogue (Ekseth et al. 2014). Proteins were considered for orthology clustering if the proteins had at least 50% identity and at least 50% overlap. To determine the orthologous groups (OGs), Markov clustering (MCL) was performed using MCL-edge (Enright et al. 2002). Genes encoding the proteins were aligned with each other within their respective OGs using MUSCLE (Edgar 2004). A super alignment was created by concatenating the aligned genes according to their position in Cft strain 03-427 if they were present in all isolates. Gaps were removed using Gblocks (Castresana 2000). Recombination events were detected in this super alignment using Gubbins (Croucher et al. 2014) with the default settings. Phylogenetic dendrograms were created using Fasttree (Price et al. 2009).

## Results and Discussion

### General Features of the *C. fetus* subsp. *testudinum* Genomes

The circular genome size of Cft strains 03-427 and SP3 is 1.78 and 1.82 Mb, respectively, which is within the known size range of *Campylobacter* (1.53–1.97 Mb) and similar to Cff strain 82-40 (1.77 Mb) (Miller 2008) (supplementary table S1, Supplementary Material online). The average G + C content of both strains is 33.1%. The Cft strain 03-427 genome is predicted to contain 1,695 putative protein-coding genes, 43 tRNA genes, and three rRNA operons. Cft strain SP3 is predicted to contain 1,767 putative protein-coding genes, 40 tRNA genes, and three rRNA operons. No obvious mobile elements or plasmids were identified in either strain. Cft strain 03-427 contained 29 variable homopolymeric GC tracts (≥8 bp; 34 total GC tracts) and strain SP3 contained 24 variable homopolymeric GC tracts (≥8 bp; 29 total GC tracts).

### Genetic Features Specific to *C. fetus* subsp. *testudinum*

**Fig. 1.**
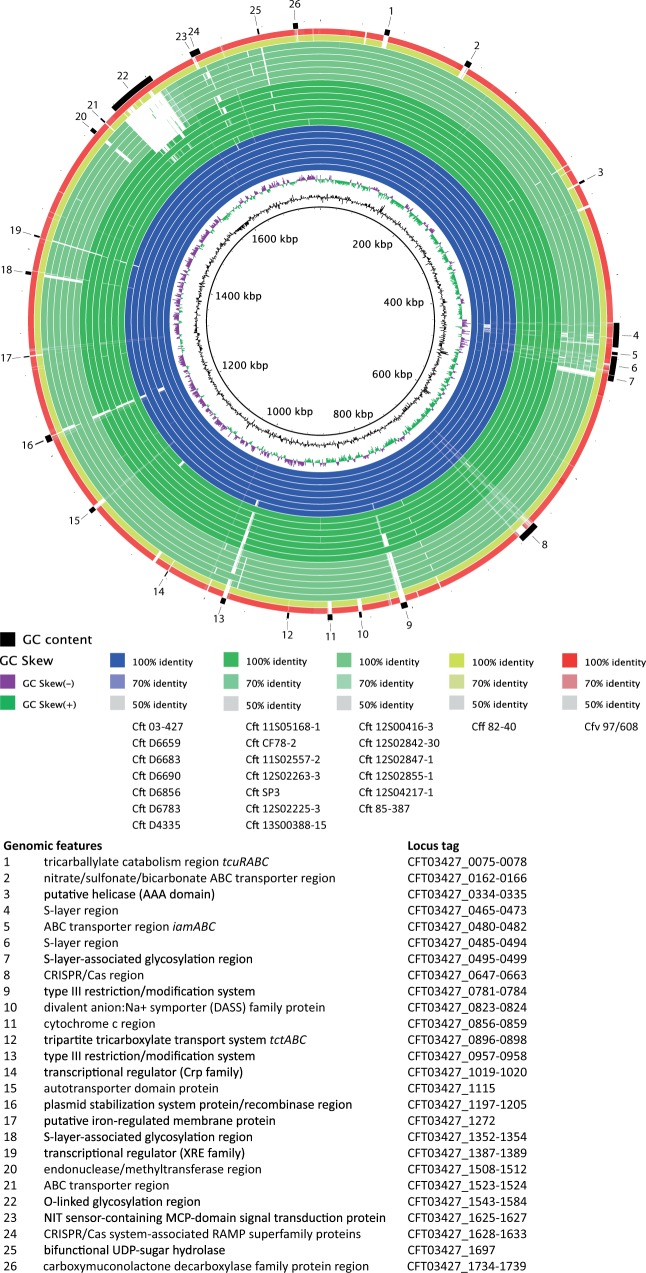
**BRIG plot of *C*. *fetus*.** BLASTN-based genomic comparison of all Cft strains, including Cft strain 03-427 (reference genome), Cff strain 82-40, and Cfv strain 97/608. Cft strains isolated from humans are shown in blue; Cft strains isolated from reptiles are shown in green, with predicted Cft *sapA* strains shown in dark green and predicted Cft *sapB* and *sapAB* strains shown in light green; Cff is shown in yellow; and Cfv is shown in red. Characteristic genomic features of *C*. *fetus* and Cft in particular have been highlighted. Features containing only hypothetical proteins are not indicated.

Several genetic features specifically present or absent from Cft were identified (supplementary table S2, Supplementary Material online). A total of 33 genes was present in all Cft strains, but absent from all Cff and Cfv strains (supplementary table S3, Supplementary Material online). The most prominent difference identified between the *C. fetus* subspecies was a putative *tcuRABC* locus (CFT03427_0075-0078), present in all Cft strains, but absent from Cff and Cfv (fig. 1). Proteins encoded by the *tcuRABC* locus have been shown to function in the catabolism of tricarballylate (a citrate analog) (Lewis et al. 2004). It has been shown that *Salmonella enterica* serovar Typhimurium strain LT2 can use tricarballylate as a carbon and energy source, which feeds directly into the citric acid cycle (Gutnick et al. 1969; Lewis et al. 2004). These results suggest that tricarballylate could potentially be used as a carbon and energy source by Cft. A BLASTP analysis revealed that this pathway was also present in *Campylobacter coli*, *Campylobacter cuniculorum*, *C*. *hyointestinalis* subsp. *lawsonii*, *C*. *iguaniorum*, and *C*. *jejuni*. In *C*. *hyointestinalis* subsp. *lawsonii*, *tcuR* was absent, whereas in both Cft and *C*. *iguaniorum* isolated from reptiles *tcuRABC* was complete. Noteworthy, *tcuRABC* appears present mainly in *Campylobacter* taxa associated with hindgut fermenting vertebrates. This suggests that the pathway is conserved in *Campylobacter* taxa inhabiting a potentially similar intestinal niche where tricarballylate is available as a carbon and energy source.

A total of 23 genes was present in all Cff and Cfv strains, but absent from all Cft strains (supplementary table S3, Supplementary Material online). Interestingly, an aspartate racemase-encoding gene (CFF8240_1412) was present in all of the Cff and Cfv strains, but absent from Cft strains. As aspartate racemase catalyzes the conversion of L-aspartate to D-aspartate, Cft is predicted to be unable to convert L-aspartate to D-aspartate. As this was a pseudogene due to a premature stop codon in Cff 04/554, but not in the other completed Cff and Cfv genomes, and this gene is located in the lipooligosaccharide (LOS) biosynthesis locus (CFF8240_1399-1414) bound by *waa* genes and containing multiple glycosyltransferases (Gilbert et al. 2008), it is likely related to LOS structure, and one might expect expression to vary depending on serotype in Cff and Cfv.

### Genetic Features Specific to *C. fetus*

Several genetic features were identified in *C*. *fetus*, but were absent from the related species *C*. *hyointestinalis* and *C*. *iguaniorum* (supplementary table S2, Supplementary Material online). In total, 65 genes were present in all *C*. *fetus* strains, but absent from both *C*. *hyointestinalis* and *C*. *iguaniorum* strains (supplementary table S3, Supplementary Material online).

Genes encoding succinyl-CoA synthetase alpha and beta subunits (*sucDC*; CFT03427_0903-0904), proline dehydrogenase (*putA*; CFT03427_1218) and sodium/proline symporter (*putP*; CFT03427_1219), hemin ABC transporter system (*chuABCD*; CFT03427_1703-1706), and serine protease (CFT03427_1733) were present in all *C*. *fetus* strains examined, but absent from its closest relatives.

In addition to catalase (CFT03427_1038), another catalase-like protein encoding region (CFT03427_1708) was present in all *C*. *fetus* strains examined. In Cff 82-40 and Cfv 97/608 however, this was a probable pseudogene. In parallel with catalase, which catalyzes the decomposition of hydrogen peroxide to water and oxygen and is present in many *Campylobacter* species, this catalase-like protein might be involved in protection against oxidative damage by reactive oxygen species.

The L-fucose permease-encoding gene *fucP* and the surrounding coding region (CFT03427_1042-1047) was present in all *C*. *fetus* strains examined, but was absent from its closest relatives *C*. *iguaniorum* and *C*. *hyointestinalis*. Low homology orthologs (57–65% identity) were identified in *C*. *coli* and *C*. *jejuni* by an online BLASTP search against the nonredundant database. In some *C*. *jejuni* strains, *fucP* and the surrounding coding region (cj0480c–cj0490) are implicated in the uptake of the sugar L-fucose, which is released from the host’s mucin glycoproteins and has been shown to be important in colonizing hosts (Muraoka and Zhang 2011; Stahl et al. 2011). Noteworthy, *fucP* and the surrounding coding region were present in *C*. *coli* showing introgression by *C*. *jejuni* genes, suggesting that the presence of this region provides *C*. *coli* an advantage in the gastrointestinal tract (Sheppard et al. 2013). Fermentation of carbohydrates is considered uncommon in *Campylobacter*, which might be related to the absence of 6-phosphofructokinase needed for glycolysis and key enzymes in the Entner-Doudoroff pathway (Parkhill et al. 2000; Kelly 2008). However, the presence of *fucP* and the surrounding coding region in *C*. *fetus* predicts that, besides amino acids and organic acids, L-fucose can be metabolized by *C*. *fetus*. In parallel with *C*. *jejuni*, this region might be involved in colonization of the host’s gastrointestinal tract.

### Prophage and Foreign DNA Defense Mechanisms

In Cft strain SP3, a 35,178 bp putative prophage was present between a leucyl tRNA and the *cas* genes of the CRISPR/Cas system. No known toxins or virulence factors were identified within this putative prophage. In Cft strain 03-427, no prophages were identified, although a region encoding hypothetical proteins with unknown function was identified in the same CRISPR/Cas region, between the leucyl tRNA and the *cas* genes. In the complete genomes of the Cff and Cfv strains examined, this location contained phage-like elements in Cff strains 82-40 and 04/554, but not in Cfv.

CRISPRs were identified in the complete genomes of Cft strains 03-427 and SP3. Six genes coding for the CRISPR-associated proteins Cas1-6 (CFT03427_0656-0663) were conserved in all 20 Cft strains examined. However, these *cas* genes were identified in only 20.5% (8/39) of the Cff and Cfv genomes (supplementary table S2, Supplementary Material online). Noteworthy, all Cfv strains were lacking the *cas* genes, while eight Cff strains, including strains 82-40, B0131, and JYCP01, which are most closely related to Cfv, did contain these genes. Although the *cas* genes were absent from Cfv strain 97/608, this strain did contain CRISPRs. The presence of CRISPRs suggests that *cas* genes may have been present, but have been lost in Cfv strain 97/608. Notably, the *cas* genes identified in *C*. *fetus* were not homologous with *cas* genes found in other *Campylobacter* species and showed highest homology with *Sulfurospirillum* and *Sulfurovum* species.

Interestingly, four additional CRISPR/Cas system-associated RAMP superfamily protein coding loci (CFT03427_1628-CFT03427_1633) were conserved in 93.2% (55/59) of the *C*. *fetus* strains (supplementary table S2, Supplementary Material online). These genes are largely confined to *C*. *fetus*, although orthologs were identified with low homology in *C*. *concisus* and *C*. *rectus*. The exact function of these proteins is unknown. No CRISPRs were identified surrounding these genes, suggesting that this cluster of genes is unlike currently known CRISPR/Cas systems.

### Virulence Determinants and Surface Structures

Most known and predicted virulence determinants, such as *cadF*, *ciaB*, and cytolethal distending toxins *cdtABC*, identified in *C*. *fetus* previously (Ali et al. 2012), were also identified in Cft. Two adjacent genes encoding a hemagglutinin/haemolysin-related protein (CFT03427_0734) and a haemolysin secretion/activation protein (CFT03427_0735) were identified in all *C*. *fetus* strains examined, but not in *C*. *hyointestinalis* and *C*. *iguaniorum*. Additionally, a patatin-like phospholipase (CFT03427_1717) was exclusively found in *C*. *fetus*. In *Pseudomonas aeruginosa*, a patatin-like protein has been linked to the development of lung injury, sepsis, and bacterial dissemination in animal models and human infections (Banerji and Flieger 2004). Type IV secretion system related gene clusters, such as *virB* genes, were commonly present in Cfv and in Cff strain 98/445 (van der Graaf-van Bloois et al. 2016), but were absent from Cft (supplementary table S2, Supplementary Material online).

One of the most prominent and distinguishing surface structures in *C*. *fetus* is the S-layer, which is associated with resistance to complement-mediated killing and is considered to be an important virulence factor (Blaser et al. 2008). The *C*. *fetus* S-layer is encoded by the *sap* locus, which contains the conserved *sapCDEF* locus and multiple copies of either *sapA* or *sapB*, and occasional *sapAB* recombinants. The completed genomes of Cft strains 03-427 and SP3 were predicted to contain eight *sapA* copies. Of all Cft strains examined, 70% (14/20) were *sapA* type, 15% (3/20) were *sapB* type, and 15% (3/20) were *sapAB* type (table 1). Remarkably, the *sapCDEF* locus was not observed in Cft strain D6683, Cff strains B0047 and S0478D, and Cfv strains 642-21, CCUG 33900, and ADRI 513 (supplementary table S2, Supplementary Material online). Considering the high variability of this region and the draft nature of the genomes, the sequence reads of these particular strains were searched for parts of these genes and were found absent in the reads as well. As these genes are essential in formation of the S-layer (Blaser et al. 2008), these particular strains may be unable to form an S-layer. The inability to form an S-layer, associated with an 8–9 kb deletion in the *sap* locus, has been shown before in spontaneous *C*. *fetus* mutants (Dworkin et al. 1995).

Adjacent to the *sap* region, 14 Cft strains contained a conserved glycosylation region associated with *sapA* (CFT03427_0495-0499), whereas the conserved glycosylation region characteristic for *sapB* (CFF04554_0484-0487), including GDP-D-mannose dehydratase-encoding *wcbK* (Kienesberger et al. 2014), was identified in six Cft strains isolated from reptiles, which were *sapB* and *sapAB* type strains (table 1). These latter strains were also missing another glycosylation region (CFT03427_1352-1354), which was also absent from all mammal-associated *sapB* type *C*. *fetus* strains, but was present in all *sapA* type *C*. *fetus* strains examined, suggesting it is associated with glycosylation in *sapA* type *C*. *fetus* (supplementary figs. S1 and S2, Supplementary Material online). The two different conserved glycosylation regions adjacent to the *sap* locus are likely involved in LPS-biosynthesis and Sap binding, as has been shown for *wcbK* in *sapB* type *C*. *fetus* (Kienesberger et al. 2014).

In seven of the Cft strains isolated from reptiles, including all predicted *sapB* and *sapAB* type strains, the region containing *flaAB* and the motility accessory factor *maf* (CFT03427_1587-1590) was highly divergent from all other *C*. *fetus* strains examined, and *flaAB* and *maf* showed highest homology with the related species *C*. *hyointestinalis* and *C*. *iguaniorum* (Gilbert, Miller, et al. 2014), suggesting recombination between Cft and a strain closely related to these taxa, which was supported by recombination analysis. Recombination of *flaAB*, possibly due to selective pressure of the host immune response, has been shown in *C*. *jejuni* (Wassenaar et al. 1995).

Remarkably, a large region (∼49 kb) adjacent to *flaAB*, encoding many O-linked glycosylation-related proteins (CFT03427_1543-1586), was conserved in all Cff/Cfv and most Cft strains examined, but was absent from the seven *flaAB* recombinant Cft strains (fig. 1). Within this region, genes encoding multiple glycosyltransferases and two asparagine synthases were identified. Of the 29 variable homopolymeric GC tracts identified within the Cft strain 03-427 genome, 12 hypervariable GC tracts (41%) were located within this glycosylation region. In *C*. *jejuni*, O-linked glycosylation is associated with flagellar assembly and function (Guerry 2007), and genes in the pseudaminic acid biosynthetic pathway (*pseB* and *pseH*) are found in and adjacent to this region. The strong association with *flaAB* in the nonrecombinant strains suggests that this region is involved in the O-linked glycosylation of flagellin. Like in the *flaAB* recombinant Cft strains, this region was missing in *C*. *iguaniorum* (Gilbert, Miller, et al. 2014). No other comparable glycosylation region was found conserved in all recombinant *flaAB* Cft strains, suggesting differential glycosylation of flagellin in these strains.

**Fig. 2.**
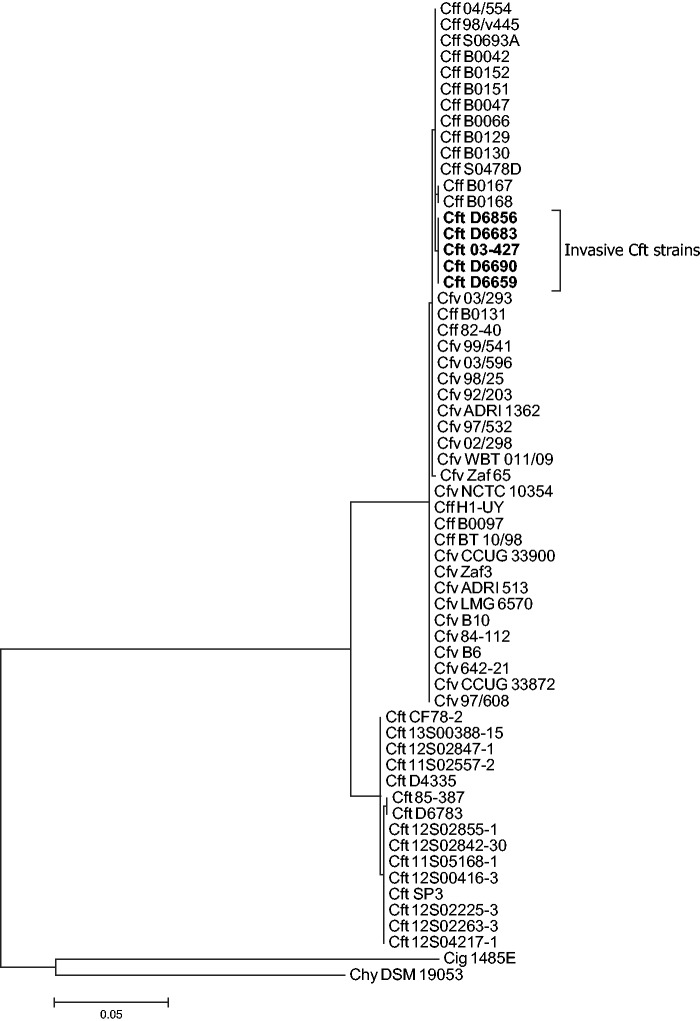
**Single-gene phylogeny of *iamA* (*mlaF*) for all strains examined.** The recombinant invasive Cft strains isolated from humans are shown in bold.

Interestingly, a region encoding ABC-transporters *iamABC* (also annotated as *mlaFED*; CFT03427_0480-0484), and showing higher than expected amino acid homology (99–100%) between all Cff and Cfv strains and human clinical Cft strains, but not reptilian Cft strains (93–97%), was observed. Recombination analysis indicated a recombination event between Cff and Cft isolated from humans. A maximum-likelihood phylogenetic dendrogram based on *iamA* (*mlaF*) illustrated this recombination event (fig. 2). This recombination was identified in 71.4% (5/7) of the Cft strains isolated from humans, but was not identified in strains isolated from reptiles. Although from different geographical origins (Dingle et al. 2010; Patrick et al. 2013), all strains with this potential recombination were closely related and belonged to the same sequence type (ST15). Notably, all these Cft strains were *atpA* (*uncA*) MLST allele type 6, whereas all other Cft strains known are MLST allele type 5 (Dingle et al. 2010; Wang et al. 2013; Gilbert, Kik, et al. 2014), which corresponds to one point mutation. A closer inspection of the clinical background of the Cft strains isolated from humans revealed that all of the recombinant strains were isolated from bile, blood, a hematoma, or pleural fluid, whereas the nonrecombinant strains were isolated from stool samples (table 1), suggesting that the recombinant strains may be invasive. Orthologs of *iamABC* are found across the *Campylobacter* genus. Notably, in all *C*. *fetus* strains examined, the *iamABC* region is conserved amidst the highly recombining *sap* region. The ABC-transporter encoded by *iamA* is considered a virulence factor associated with invasion in *C*. *jejuni* (Carvalho et al. 2001). Most *C*. *fetus* infections in humans are systemic or have a systemic component and the ratio of systemic infection to diarrheal illnesses for *C*. *fetus* is much higher than for *C*. *jejuni*, indicating a propensity for invasive disease compared with *C*. *jejuni* (Blaser et al. 2008; Patrick et al. 2013; Wagenaar et al. 2014). The mechanisms of invasion in *C*. *fetus* are poorly understood; however, considering both mammal- and reptile-associated *C*. *fetus* with recombinant *iamABC* show invasion, the *iamABC* region may have a similar function in invasive *C*. *fetus* strains.

### Campylobacter fetus *Phylogeny*

**Fig. 3.**
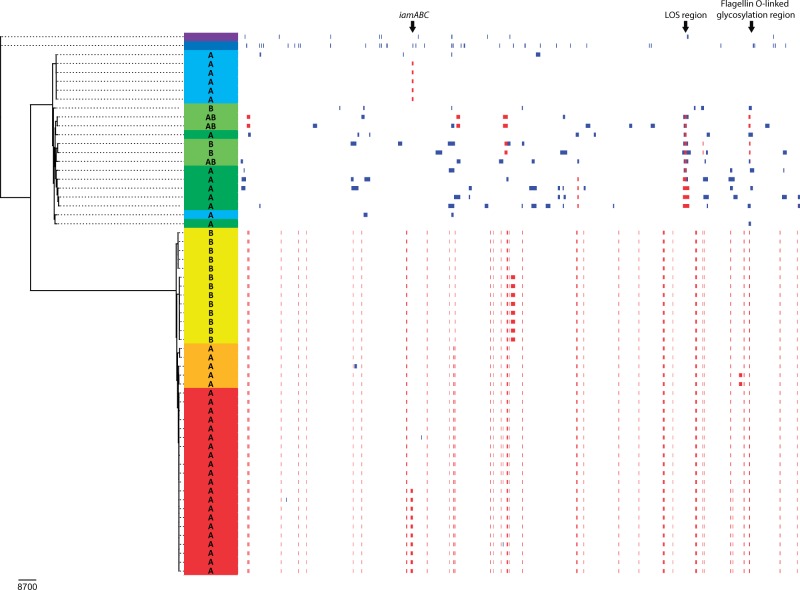
**Phylogenetic reconstruction of the *C*. *fetus* clade based on Gubbins.** Recombination regions within the 781,293 nt gapless core genome alignment are indicated in red (similar recombination region in multiple strains) or blue (unique recombination region). Species and subspecies from top to bottom: *C*. *iguaniorum*, purple; *C*. *hyointestinalis*, dark blue; *C*. *fetus* subsp. *testudinum* (human strains), light blue; *C*. *fetus* subsp. *testudinum* (reptilian strains), dark green (*sapA* strains) or light green (*sapB* and *sapAB* strains); *C*. *fetus* subsp. *fetus* (based on genotype), yellow (*sapB* strains) or orange (*sapA* strains); *C*. *fetus* subsp. *venerealis* (based on genotype), red. For *C*. *fetus*, the *sap* type of the corresponding strains is indicated with A, B, or AB. To increase the intraspecies resolution for *C*. *fetus*, the branches of the dendrogram are truncated for *C*. *hyointestinalis* and *C*. *iguaniorum*. Recombination regions of interest have been highlighted.

*Campylobacter fetus* is generally considered a genetically coherent species with low genetic diversity compared with some other *Campylobacter* species (van Bergen et al. 2005). A phylogenetic reconstruction accounting for the effects of homologous recombination was performed for *C*. *fetus* and the most closely related species, based on a 781,293 nt gapless alignment (fig. 3). Mammal- and reptile-associated *C*. *fetus* formed two clearly separated clades. This is in line with earlier observations that mammal- and reptile-associated *C*. *fetus* form two distinct clades with an average amino acid identity of 95–96% and supports the description of *C*. *fetus* subsp. *testudinum* as a novel subspecies (Fitzgerald et al. 2014). The core genome phylogeny confirmed that genetic diversity was higher among Cft than among Cff and Cfv. A shorter branch length suggests that Cft is more closely related to the last common ancestor. Genetic diversity was higher in Cft isolated from reptiles, which may be related to a larger diversity among the sampled reptile population. All invasive Cft strains isolated from humans showed little genetic diversity among each other and formed a separate lineage together with Cft strain D6783, distinct from the Cft strains isolated from reptiles, confirming earlier observations based on MLST and AFLP (Dingle et al. 2010; Fitzgerald et al. 2014).

The number of single nucleotide polymorphisms (SNPs) detected inside recombinations was higher in Cft, whereas the number of SNPs outside recombinations was lower in Cft, compared with Cff and Cfv (table 2). Also the ratio of base substitutions predicted to have been imported through recombination to those occurring through point mutation (r/m) and the ratio of the number of recombination events to point mutations (rho/theta) were higher in Cft. This indicates that mutation through recombination is more important than mutation through point mutation in Cft, compared with Cff and Cfv, based on the strains in this study. Predicted recombinant regions were subdivided into regions shared by two or more strains, indicated by red blocks, or unique regions, indicated by blue blocks (fig. 3). More shared recombination events were detected in Cff/Cfv than in Cft. In contrast, more unique recombination events were detected in Cft strains isolated from reptiles. However, the invasive Cft strains isolated from humans and Cft strain D6783 showed no unique recombination events, which supports the close genetic relationship of these strains.

**Table 2:** Summary of the Recombination Analysis for Cft and Cff and Cfv

	Cft	**Cff and Cfv**
Total SNPs	25,562	62,228
Number of SNPs inside recombinations	7,970	3,103
Number of SNPs outside recombinations	17,592	59,125
Number of recombination blocks	107	56
Bases in recombinations	497,384	820,273
r/m	0.453	0.052
rho/theta	0.006	0.001
Genome length	781,293	781,293

Note.—The ratio of base substitutions predicted to have been imported through recombination to those occurring through point mutation is indicated by r/m. The ratio of the number of recombination events to point mutations is indicated by rho/theta.

A clear association between phylogeny and *sap* type was observed in Cff and Cfv, with *sapA* and *sapB* type strains forming separate clusters (fig. 3). This confirms the previously shown correlation between MLST sequence types and *sap* types (van Bergen et al. 2005). However, in Cft no association between phylogeny and *sap* type was observed, which may be explained by the larger influence of recombination in Cft. The presence of *sapAB* recombinant Cft strains also suggests that the *sap* locus is less conserved in Cft. Reptile-associated Cft can be *sapA*, *sapB*, or *sapAB*, which is in contrast to previous studies (Tu et al. 2005; Dingle et al. 2010). The identification of these *sap* types in genetically diverse Cft strains shows that these *sap* types are widespread in Cft and are present in both mammal- and reptile-associated *C*. *fetus*. In contrast to previous studies (Tu et al. 2005; Kienesberger et al. 2014), this suggests that both *sap* types were likely present before the mammal- and reptile-associated *C*. *fetus* lineages diverged, although it cannot be excluded that *sap* types recombined between mammal and reptile-associated *C*. *fetus* at a later stage. The presence of *sapAB* chimeras confirms that recombination of these different *sap* types occurs. As such, the diversity of the *sap* locus, which encodes a surface antigen under diversifying selection imposed by the host immune response, may hamper evolutionary assumptions.

Despite the different, well-separated niches, and genome-wide genetic divergence, mammal- and reptile-associated *C*. *fetus* are similar in overall gene content and synteny. In this study, only a few recombination events between mammal- and reptile-associated *C*. *fetus* were observed, suggesting that recombination between mammal- and reptile-associated *C*. *fetus* can be considered rare and that effective barriers to recombination exist, likely due to the separate host reservoirs, although other factors, such as genetic divergence and other intrinsic factors inhibiting recombination cannot be excluded. This is consistent with allopatric speciation. In contrast, Cff and Cfv show a nearly identical core proteome (van der Graaf-van Bloois et al. 2014), and different niche preferences are potentially associated with laterally acquired elements in Cfv (Ali et al. 2012; Kienesberger et al. 2014). Indeed, within *C*. *fetus*, Cfv showed the largest (accessory) genomes. Based on the high genetic similarity between Cff and Cfv, and the lack of a clear boundary between these subspecies, the validity of these subspecies has been questioned (van Bergen et al. 2008; van der Graaf-van Bloois et al. 2014). Nevertheless, Cff and Cfv do show small but consistent genetic differences based on phylogenetic analyses, indicating genetic divergence of these two lineages, which could be explained by an ecological barrier in which Cff and Cfv do not or barely recombine despite a high degree of niche overlap, as has been demonstrated for *C*. *jejuni* (Sheppard et al. 2014). Alternatively, the low genetic diversity of *C*. *fetus* may be explained by a lack of natural competence, as observed in vitro (Tu et al. 2003; Kienesberger et al. 2007, 2014), or, conversely, by a high rate of homologous recombination within the different *C*. *fetus* lineages, maintaining lineage coherence. With the advent of whole-genome sequencing, recombination is considered more common than previously assumed and observed clonality does often not exclude recombination (Feil et al. 2001), as was identified in this study for *C. fetus*.

## Conclusions

The genomes of *C*. *fetus* subsp. *testudinum* in this study show high conservation in gene content and synteny compared with other *C*. *fetus* subspecies. Although the similarity between reptile- and mammal-associated *C*. *fetus* is high, the genomes are clearly divergent in overall sequence identity and in gene content.

Most notable features shared by the majority of *C*. *fetus* strains in this study are the S-layer, a CRISPR/Cas system and CRISPR/Cas system-associated RAMP superfamily proteins unique for *Campylobacter*. Several genomic differences were observed between mammal- and reptile-associated *C*. *fetus*, of which the presence of a putative tricarballylate catabolism pathway in Cft is most notable. In contrast to earlier observations, *C*. *fetus* subsp. *testudinum* can contain *sapA*, *sapB*, or *sapAB*. In seven Cft strains isolated from reptiles the region containing *flaAB* and *maf* was highly divergent. These strains were lacking a large adjacent glycosylation region conserved in all other *C*. *fetus* strains.

Recombination between mammal- and reptile-associated *C*. *fetus* seems rare, indicating effective barriers to recombination between these two divergent lineages, likely due to the separate host reservoirs, consistent with allopatric speciation. Nevertheless, a recombination event of *iamABC* between mammal-associated *C*. *fetus* and Cft strains isolated from humans was observed, which is associated with invasion in humans.

The whole-genome sequences of reptile-associated *C*. *fetus* subsp. *testudinum* provide a better understanding of *C*. *fetus* as a species and genomic features associated with subspecies, host type, and virulence, and provide further insights in *C. fetus* biology and evolution.

## Supplementary Material

Supplementary figures S1 and S2 and tables S1–S3 are available at *Genome Biology and Evolution* online (http://www.gbe.oxfordjournals.org/).

Supplementary Data
