# Supplementary material for: Comparative Genomics of Campylobacter fetus from Reptiles and Mammals Reveals Divergent Evolution in Host-Associated Lineages
Source: Genome Biol Evol. 2016 Jun 22;8(6):2006–19. doi: 10.1093/gbe/evw146 (PMC4943207; doi:10.1093/gbe/evw146)
Supplement: Supplementary Data [file supp_8_6_2006__index.html]

Comparative Genomics of Campylobacter fetus from Reptiles and Mammals Reveals Divergent Evolution in Host-Associated Lineages — Supplementary Data 

# Comparative Genomics of *Campylobacter fetus* from Reptiles and Mammals Reveals Divergent Evolution in Host-Associated Lineages

## Supplementary Data

files

- Supplementary Data - zip file
