# Supplementary material for: Comparative Genomics of Campylobacter fetus from Reptiles and Mammals Reveals Divergent Evolution in Host-Associated Lineages
Source: Genome Biol Evol. 2016 Jun 22;8(6):2006–19. doi: 10.1093/gbe/evw146 (PMC4943207; doi:10.1093/gbe/evw146)
Supplement: Supplementary Data [file supp_evw146_suppl_data.zip › Figure_S1.pdf]

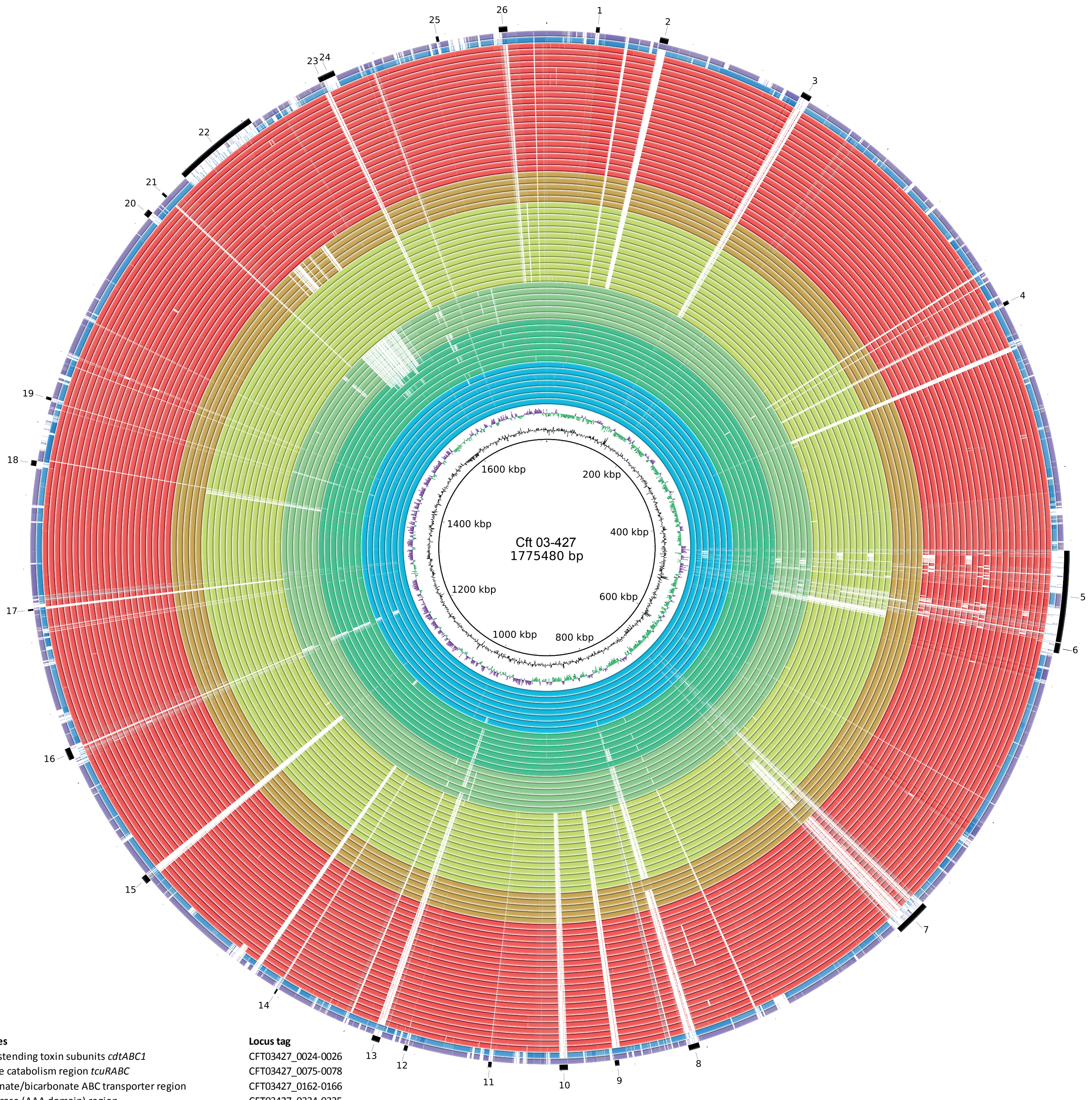

Genomic features	Locus tag
1 cytolethal distending toxin subunits *cdtABC1*	CFT03427_0024-0026
2 tricarballylate catabolism region *tcuRABC*	CFT03427_0075-0078
3 nitrate/sulfonate/bicarbonate ABC transporter region	CFT03427_0162-0166
4 putative helicase (AAA domain) region	CFT03427_0334-0335
5 S-layer region	CFT03427_0465-0494
6 S-layer-associated glycosylation region	CFT03427_0495-0499
7 CRISPR/Cas region	CFT03427_0647-0663
8 type III restriction/modification system	CFT03427_0781-0784
9 divalent anion:Na+ symporter (DASS) family protein	CFT03427_0823-0824
10 cytochrome C region	CFT03427_0856-0859
11 tripartite tricarboxylate transport system *tctABC*	CFT03427_0896-0898
12 cytolethal distending toxin subunits *cdtABC2*	CFT03427_0944-0946
13 type III restriction/modification system	CFT03427_0957-0958
14 transcriptional regulator (Crp family)	CFT03427_1019-1020
15 autotransporter domain protein	CFT03427_1115
16 plasmid stabilization system protein/recombinase region	CFT03427_1197-1205
17 putative iron-regulated membrane protein	CFT03427_1272
18 S-layer-associated glycosylation region	CFT03427_1352-1354
19 transcriptional regulator (XRE family)	CFT03427_1387-1389
20 endonuclease/methyltransferase region	CFT03427_1508-1512
21 ABC transporter region	CFT03427_1523-1524
22 O-linked glycosylation region	CFT03427_1543-1584
23 NIT sensor-containing MCP-domain signal transduction protein	CFT03427_1625-1627
24 CRISPR/Cas system-associated RAMP superfamily protein region	CFT03427_1628-1633
25 bifunctional UDP-sugar hydrolase	CFT03427_1697
26 carboxymuconolactone decarboxylase family protein region	CFT03427_1734-1739

Cft 03-427	Cft 11S05168-1	Cft 12S00416-3	Cff 98v445	Cff B0097	Cfv 642-21	Chy DSM 19053	Cig 1485E
Cft D6659	Cft CF78-2	Cft 12S02842-30	Cff B0066	Cff BT 10/98	Cfv ADRI 513		
Cft D6683	Cft 11S02557-2	Cft 12S02847-1	Cff B0130	Cff H1-UY	Cfv CCUG 33872		
Cft D6690	Cft 12S02263-3	Cft 12S02855-1	Cff B0129	Cff 82-40	Cfv Zaf 3		
Cft D6856	Cft SP3	Cft 12S04217-1	Cff S0478D	Cff B0131	Cfv 97/608		
Cft D6783	Cft 12S02225-3	Cft 85-387	Cff 04/554		Cfv 84-112		
Cft D4335	Cft 13S00388-15		Cff S0693A		Cfv B10		
			Cff B0167		Cfv CCUG 33900		
			Cff B0168		Cfv LMG 6570		
			Cff B0047		Cfv B6		
			Cff B0151		Cfv NCTC 10354		
			Cff B0152		Cfv WBT 011/09		
			Cff B0042		Cfv Zaf 65		
					Cfv 03/293		
					Cfv 97/532		
					Cfv 92/203		
					Cfv 03/596		
					Cfv 98/25		
					Cfv 99/541		
					Cfv ADRI 1362		
					Cfv 02/298		
