# Supplementary material for: Comparative Genomics of Campylobacter fetus from Reptiles and Mammals Reveals Divergent Evolution in Host-Associated Lineages
Source: Genome Biol Evol. 2016 Jun 22;8(6):2006–19. doi: 10.1093/gbe/evw146 (PMC4943207; doi:10.1093/gbe/evw146)
Supplement: Supplementary Data [file supp_evw146_suppl_data.zip › Figure_S2.pdf]

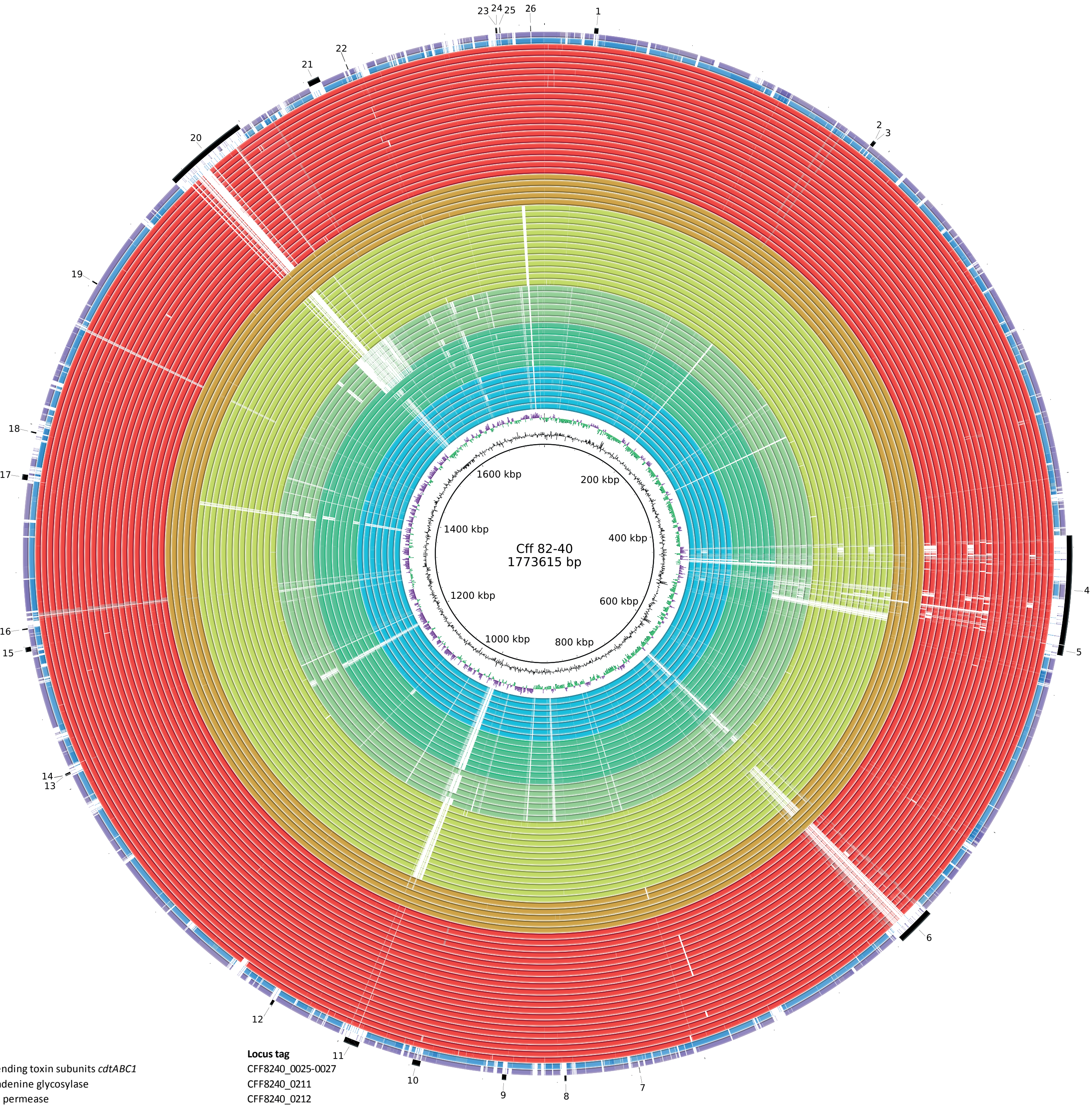


| Genomic features | Locus tag |
|---|---|
| 1 cytolethal distending toxin subunits *cdtABC1* | CFF8240_0025-0027 |
| 2 DNA-3-methyladenine glycosylase | CFF8240_0211 |
| 3 xanthine/uracil permease | CFF8240_0212 |
| 4 S-layer region | CFF8240_0456-0490 |
| 5 S-layer-associated glycosylation region | CFF8240_0491-0495 |
| 6 CRISPR/Cas region | CFF8240_0644-0666 |
| 7 conserved hypothetical protein | CFF8240_0825 |
| 8 membrane protein | CFF8240_0872 |
| 9 tripartite tricarboxylate transport system *tctABC* | CFF8240_0902-0904 |
| 10 cytolethal distending toxin subunits *cdtABC2/3* | CFF8240_0951-0957 |
| 11 type I restriction/modification system | CFF8240_0985-0991 |
| 12 transporter, sodium:sulfate symporter family | CFF8240_1040 |
| 13 transcriptional regulator, HxlR family | CFF8240_1224 |
| 14 putative NADH-flavin reductase | CFF8240_1225 |
| 15 ATP-binding protein region | CFF8240_1289-1291 |
| 16 putative azoreductase | CFF8240_1299 |
| 17 S-layer-associated glycosylation region | CFF8240_1389-1391 |
| 18 aspartate racemase | CFF8240_1412 |
| 19 conserved hypothetical protein (MOSC domain) | CFF8240_1502 |
| 20 O-linked glycosylation region | CFF8240_1584-1632 |
| 21 CRISPR/Cas system-associated RAMP superfamily proteins | CFF8240_1674-1679 |
| 22 membrane protein | CFF8240_1700 |
| 23 putative NADPH-quinone reductase (modulator of drug activity B) | CFF8240_1794 |
| 24 flavodoxin | CFF8240_1795 |
| 25 conserved hypothetical protein | CFF8240_1799 |
| 26 TfoX domain-containing protein | CFF8240_1817 |

| | | | | | | | |
|---|---|---|---|---|---|---|---|
| Cft 03-427 | Cft 11S05168-1 | Cft 12S00416-3 | Cff 98v445 | Cff B0097 | Cfv 642-21 | Chy DSM 19053 | Cig 1485E |
| Cft D6659 | Cft CF78-2 | Cft 12S02842-30 | Cff B0066 | Cff BT 10/98 | Cfv ADRI 513 | | |
| Cft D6683 | Cft 11S02557-2 | Cft 12S02847-1 | Cff B0130 | Cff H1-UY | Cfv CCUG 33872 | | |
| Cft D6690 | Cft 12S02263-3 | Cft 12S02855-1 | Cff B0129 | Cff 82-40 | Cfv Zaf 3 | | |
| Cft D6856 | Cft SP3 | Cft 12S04217-1 | Cff S0478D | Cff B0131 | Cfv 97/608 | | |
| Cft D6783 | Cft 12S02225-3 | Cft 85-387 | Cff 04/554 | | Cfv 84-112 | | |
| Cft D4335 | Cft 13S00388-15 | | Cff S0693A | | Cfv B10 | | |
| | | | Cff B0167 | | Cfv CCUG 33900 | | |
| | | | Cff B0168 | | Cfv LMG 6570 | | |
| | | | Cff B0047 | | Cfv B6 | | |
| | | | Cff B0151 | | Cfv NCTC 10354 | | |
| | | | Cff B0152 | | Cfv WBT 011/09 | | |
| | | | Cff B0042 | | Cfv Zaf 65 | | |
| | | | | | Cfv 03/293 | | |
| | | | | | Cfv 97/532 | | |
| | | | | | Cfv 92/203 | | |
| | | | | | Cfv 03/596 | | |
| | | | | | Cfv 98/25 | | |
| | | | | | Cfv 99/541 | | |
| | | | | | Cfv ADRI 1362 | | |
| | | | | | Cfv 02/298 | | |
